# One-Size-Fits-All Policies Are Unacceptable: A Sustainable Management and Decision-Making Model for Schools in the Post-COVID-19 Era

**DOI:** 10.3390/ijerph19105913

**Published:** 2022-05-12

**Authors:** Cunwei Yang, Weiqing Wang, Fengying Li, Degang Yang

**Affiliations:** 1 Department of Computer Science and Technology, Southwest University, Chongqing 402460, China; yangcunwei@email.swu.edu.cn; 2 Department of Management Science and Engineering, Southwest University, Chongqing 402460, China; 3 Department of Environmental Science, Nanjing University of Information Science and Technology, Nanjing 210044, China; echobdau@nuist.edu.cn; 4 State Key Laboratory of Desert and Oasis Ecology, Xinjiang Institute of Ecology and Geography, Chinese Academy of Sciences, Urumqi 830011, China; dgyang@ms.xjb.ac.cn; 5 The Graduate School, University of Chinese Academy of Sciences, Beijing 100049, China

**Keywords:** management science, epidemiology, public health policies, COVID-19, decision-making

## Abstract

This paper proposes a sustainable management and decision-making model for COVID-19 control in schools, which makes improvements to current policies and strategies. It is not a case study of any specific school or country. The term one-size-fits-all has two meanings: being blind to the pandemic, and conducting inflexible and harsh policies. The former strategy leads to more casualties and does potential harm to children. Conversely, under long-lasting strict policies, people feel exhausted. Therefore, some administrators pretend that they are working hard for COVID-19 control, and people pretend to follow pandemic control rules. The proposed model helps to alleviate these problems and improve management efficiency. A customized queue model is introduced to control social gatherings. An indoor–outdoor tracking system is established. Based on tracing data, we can assess people’s infection risk, and allocate medical resources more effectively in case of emergency. We consider both social and technical feasibility. Test results demonstrate the improvements and effectiveness of the model. In conclusion, the model has patched up certain one-size-fits-all strategies to balance pandemic control and normal life.

## 1. Introduction

As a result of the worldwide spread of SARS-CoV-2, coronavirus disease 2019 (COVID-19) has evolved into a persistent pandemic [1]. SARS-CoV-2 is one of the most contagious viruses, which has a large number of transmission routes: respiratory droplets from coughs and sneezes, contact via contaminated objects, aerosols, etc. Some transmission routes are even unknown [2]. Some variants of SARS-CoV-2 have a long incubation period, even sometimes with asymptomatic infections. The novel virus impacts greatly on public health [3], the global economy [4], societies [5], etc. According to the current situation, the pandemic will probably last a long time and continue influencing the world [6]. Compared to Middle East respiratory syndrome (MERS) [7], Ebola [8], Marburg [9], or other infectious diseases with a high death rate, the survival rate of COVID-19-positive individuals is relatively higher [10]. However, due to the extreme infectivity of SARS-CoV-2, the number of infected people will be very large, resulting in a number of casualties [11]. Especially when there is a medical resources panic squeeze, the death rate will rise drastically. A case in point is the situation in Wuhan in 2019 [12].

This paper specifies COVID-19 prevention and control in schools. Schools can be seen as large, densely populated places. Classrooms, conference halls, canteens, etc., are always filled with people. Hence, once a person is infected, he may unconsciously infect a large crowd of people [13]. In schools, it is difficult to find a large number of rooms for quarantine within a short period. Logistical support for infected people is also extremely difficult. The term one-size-fits-all refers to two kinds of measures: being blind to the pandemic, and blocking the campus all the time and conducting harsh strategies [14].

For human beings, although numerous papers on COVID-19 have been published, studies of this novel virus are still inadequate. The RNA virus is unstable, and its various variants continue to emerge. Although adolescents are stronger than older adults, which means the mortality of younger people is lower than the aged, people with COVID-19-related symptoms are unable to do their work well. Additionally, potential sequelae may heavily influence the next generation [15].

Some schools have adopted strict lockdown policies since COVID-19. Students cannot go out de facto and they must stay at school all the time. It hinders social contact, internships, academic conferences, etc., causing a lot of mental pressure on students [16]. The primitive purpose of COVID-19 prevention and control is to guarantee our social order and normal life, but sometimes extreme and costly lockdowns cannot ensure safety, instead, leading to other losses. Therefore, in this paper, we do not suggest one-size-fits-all solutions. Neither ignorance of the pandemic nor persistent extreme lockdown is reasonable.

From the perspective of epidemiology, regardless of the viruses that cause infectious diseases, effective measures to prevent them are clear, such as controlling the source of infection, cutting off the route of transmission, and protecting uninfected people [17]. Hence, current strategies and policies are an attempt to implement these ideas.

In some schools, students and faculties are asked to report their physical condition and accurate location to administrators every day. If an individual has been to a place with COVID-19 cases, or has COVID-19-related symptoms, he will be isolated. People are asked to maintain enough distance, e.g., students cannot gather together to hold a party. Once COVID-19 cases are found in schools, all engaged people must be isolated. They cannot go out until they are confirmed COVID-19-negative. In addition, random inspections for nucleic acid tests are usual, which can help to find out if there are asymptomatic patients in the school.

Nevertheless, many details are not clear, which means some strategies are very fuzzy and inaccurate. Although we spend a lot of manpower and material resources, there will be COVID-19 outbreaks in schools sometimes. A case in point is a recent COVID-19 outbreak in a university in Jilin Province, China [18]. In fact, students in this university cannot leave campus easily in daily life. Despite a lack of social gatherings, vaccines, daily physical condition reports, etc., a number of students became infected. At first, medical resources were insufficient (medicines, quarantines, test kits, etc.), and the management seemed a bit chaotic. The managers tried hard, but they still faced difficulties in determining who should have higher priority to access medical resources, who needs to be isolated in quarantine, how to guarantee daily needs of students, etc.

The war against COVID-19 is protracted, and it is difficult for us to anticipate the end of COVID-19 based on the status quo. Persistent unsustainable and costly lockdowns will make individuals tired and annoyed, which may in turn cause serious dilution of the prevention effect. For schools, which are considered enclosed spaces, once there is negligence in epidemic prevention, the consequences will be very bad. Therefore, we need to put forward a sustainable management model for COVID-19 control in schools. The term sustainable means this model does not cost too much but improves accuracy and efficiency against the backdrop of the lasting pandemic.

In the information age, prevention and control of COVID-19 in schools could be more intelligent and precise. Normal life needs to be balanced with disease prevention and control. The real value of science and technology is in improving quality of life [19]. In this paper, we first review related studies which aim to combat the virus. We clarify the basic principles, which are scientific evidence to design the model. We use computational and mathematical models to obtain and analyze data. Data from Internet of Things (IoT) are fundamental to providing necessary tracking data for decision-making. We then simulate and test our methods to demonstrate the advantages of our model. In general, though no one can guarantee to stop the virus, we can minimize the risk and influence using this model in schools. To sum up, in this paper, we have addressed the following issues:

(1) How to gather tracking data in schools for further decision-making.
(2) What queueing algorithm can be used to control social gatherings?
(3) How to allocate limited medical resources in case of emergency.
(4) Is the model more feasible in practice compared to current means?

## 2. Related Work

A number of researchers are conducting research on COVID-19 prevention and control from their own perspective. Samui et al. [20] designed a mathematical model for COVID-19 transmission dynamics. Zhou et al. [21] studied the correlations between rare disasters, macroeconomic policy, and the exchange rate in the pandemic era. Zoabi et al. [22] built a machine learning-based model for COVID-19 diagnosis. Lin et al. [23] highlighted the use of Building Information Modeling (BIM) in hospitals during the pandemic. Lanera et al. [24] started a COVID-19ita project to develop a public open-source tool set to offer timely, updated information about the pandemic’s evolution. COVID-19 is a perplexing conundrum, but it is still controllable and preventable. Hence, exploring a sustainable model for COVID-19 control in schools is worthwhile.

Evidence has demonstrated that COVID-19 is not just a simple flu [25], which means special interventions are always required. Schools, where young people gather, deserve more attention. Similar to other infectious diseases, besides specific medicine and vaccines, common and routine ways to prevent COVID-19 such as controlling social gatherings, protecting vulnerable people, and isolating infected ones can still function well [26]. COVID-19 prevention is not only a medical problem, but also a social issue [27]. Some strict policies are effective in epidemiology, but hard to conduct in practice (e.g., blocking a city over and over again). D’Angelo et al. [28] focus on accurate COVID-19 handling measures. Therefore, designs and strategies using various technologies in this paper are implementations of current pandemic prevention guidelines in medicine.

It is important to confirm someone’s trajectory, especially in case he is considered infected [29]. COVID-19-positive individuals will unconsciously pollute the air [30], object surfaces [31], water [32], etc. Thus, people who have passed through contaminated areas may become infected. Compared to outdoor environments, indoor environments (e.g., canteens) are places with a higher infection risk [33]. For an indoor place where many people come and go, it is required to deploy an indoor tracking system to trace everyone, and then, in case of emergency, provide data to analyze people’s infection risk. In this case, an indoor positioning system should be introduced.

Indoor positioning has been a hot topic for several years, and lots of algorithms and hardware are proposed [34]. Although there exist a number of choices for indoor positioning, we should consider the feasibility in schools. Administrators and students will not be interested in a system which requires specific devices and high costs. For outdoor tracking, a satellite-based system is appropriate, such as Galileo, GPS, and GLONASS [35]. Outdoor positioning applications are more well-developed, and all smart phones support outdoor positioning technologies from a hardware level [36] to a software level [37]. Since satellite signals are not able to penetrate obstacles in an indoor environment [38], indoor positioning technology needs to be introduced for tracking individuals in indoor environments. Prevailing indoor positioning hardware such as Bluetooth Low Energy (BLE) [39], Radio Frequency Identification (RFID) [40], and ZigBee [41] are based on wireless networks. Though Ultra-Wideband (UWB) can provide high-accuracy positioning services [42], people are reluctant to bring specific hardware (most mobile phones do not support UWB) [43]. Other devices, such as Wi-Fi Access Points (APs) which require external power supply [44], ZigBee which is relatively expensive and requires requirements for channel bandwidth [45], and geomagnetic sensors which have a higher technical threshold [46], are not so appropriate for large promotion in schools. BLE-based technology, iBeacon, which was released by Apple, is the fundamental hardware for indoor positioning [47].

We hereby divide indoor positioning methods into two categories: measurement-based and fingerprint-based methods. Time of Arrival (TOA) [48], Time Difference of Arrival (TDOA) [49], Angle of Arrival (AOA) [50], etc., are typical measurement-based methods. Although these methods can provide precise positioning results with optimization, they have a strict requirement for hardware and the environment. Thus, fingerprint-based methods are introduced. A position, $P\left(a,b\right)$, is associated with a specific fingerprint. A fingerprint is a unique feature of a position, and any data which can distinguish a position can be used as fingerprints [51]. In this paper, we chose Received Signal Strength Indicator (RSSI) as fingerprints. In the offline stage, a fingerprint database is built and a real-time fingerprint is sent to the database to obtain a predicted location in the online stage [52].

For COVID-19 tracking, the requirement for accuracy is not very high. For example, whether a person is 10 or 40 cm away from the patient, he should be considered to be at great risk of infection. Therefore, there is no need to use a complex and intricate matching model, which requires much time and cost for debugging. Support Vector Machine (SVM), Long Short-Term Memory (LSTM) [53], Multilayer Perceptron (MLP), etc., are more complex than K-Nearest Neighbors (KNN) and its variants [54]. KNN-based models can be widely used, especially for schools.

In addition, our risk assessment and medical resources allocation method also refers to some existing policies [55]. Queueing theory also plays an important role in avoiding social gatherings [56].

In brief, technologies are thriving, and COVID-19 control rules are clear, but the combination of them should be studied further.

## 3. Methods

No matter what infectious viruses cause a pandemic, there exist common ways to stop the spread, or at least, reduce the loss. We could conclude that there are three main means of pandemic control: controlling the source of infection, cutting off the route of transmission, and protecting vulnerable people. These ways are closely related. If we do not perform well in any link, there will be a serious dilution of the effect of pandemic handling. Controlling social gatherings helps to reduce the number of people who have close contact with patients. Outdoor–indoor tracing helps to find out people having potential contacts with infected people, stopping the continuous virus spread. Based on tracing data, we can be aware of individuals having a higher infection risk, and then, if medical resources are quite insufficient, it is acceptable to allocate resources to these people first. Thus, the proposed methods are interrelated, and they are not independent ideas.

### 3.1. Social Gathering Control

Students and faculties are asked to keep a social distance and avoid gatherings, which are primitive but effective. These measures are not implemented very well. Crowds will not follow these rules without reasonable management and guidance. Canteens, bathrooms, water rooms, etc., are enclosed spaces in schools. Before COVID-19, these public places were always filled with people. During the pandemic time, we should control the stream of people. Unlike other situations (e.g., airports), the strategy must not be radical and aggressive. Otherwise, students will not become accustomed to or even resist it, making this measure difficult to implement.

Since individuals are not encouraged to enter these places freely, there must be a strategy for booking and waiting. Thus, a customized queueing model is introduced in this section.

We use an example of a canteen to explain the queueing model. Canteens in a school are always crowded, leading to much infection risk. The number of people allowed to eat in a canteen at the same time is also limited. To simplify, let us suppose that a canteen can only hold 1 person. There are 4 people (A, B, C, and D) attempting to go into the canteen (Table 1). The time unit in this section is minutes, by default.

At 10:25, B attempts to enter the area, however A has occupied the position, which means B has to wait until 10:30. At 10:30, $waiting\;time\;\left(w\right)$ of B is 5 min (10:30−10:25=5 min). The field p is the predicted staying time. The indicator $response\;ratio\;\left(rr\right)$ is defined as:

(1) $$rr=\left(w+p\right)/p.$$

When there exists a vacancy, the individual with higher rr has the higher priority to enter the area. Generally, a person with a shorter predicted staying time and a longer waiting time is expected to access the resource earlier.

Individuals need to stand in a queue if their requests cannot be immediately satisfied. From the perspective of pandemic prevention, we expect that the total waiting time will be minimal. A simple Short Job First (SJF) strategy can meet the requirements. The student who plans to stay for less time will have higher priority. However, it is obvious that this method is very unfair to those who need to stay longer. For example, in a canteen, a person may have to wait for a long time because he often eats slowly.

Conversely, in practice, First Come First Serve (FCFS) is a common model. It is easier to make students agree with the model because it is fair intuitively. However, considering the current situation in schools, it may cause more social gatherings and the total waiting time could be very long in case many people require lots of time. This model makes no prominent improvement for COVID-19 control. Thus, the proposed method is the combination of these two algorithms.

The indicator rr considers both people’s willingness and COVID-19 control. We can also fix the indicator further. People may provide an unreasonable predicted staying time to obtain higher priority. If people have already been waiting for too long, we do not encourage them to keep waiting. Therefore, to meet the requirement, the parameters in rr could be modified (Equation (2)):

(2) $$rr=\left(f\left(w\right)+g\left(p\right)\right)/g\left(p\right)=1+f\left(w\right)/g\left(p\right).$$

Here is an example to modify the model. We hereby think requests for staying in a canteen for less than 1 min are “unreasonable”. Therefore, when p<1, $g\left(p\right)$ remains constant ($\frac{\partial rr}{\partial p}=0$). With the increase of p, rr decreases gradually. When p>10, we think the staying time p is too long, and the reduction rate of rr can be greater ($\frac{\partial rr}{\partial p}<0$, $\frac{\partial^{2}g}{\partial p^{2}}>0$).

**Figure 1:**
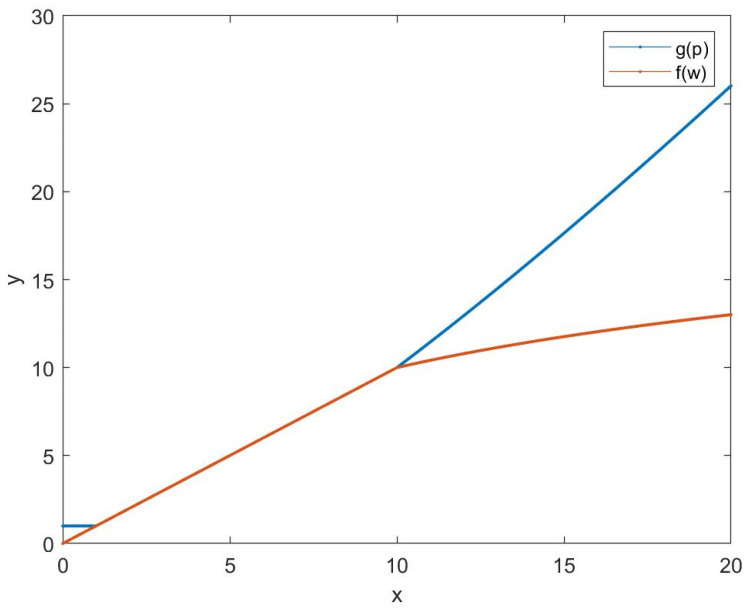
The visualization of $g\left(p\right)$ and $f\left(w\right)$.

The longer the waiting time, the higher the priority of obtaining permission. However, people waiting too long (in this case, we set the threshold value as 10 min) have a higher probability of becoming infected. Thus, when w>10 (it suggests that there are many people waiting), the increase of w will not significantly influence rr ($\frac{\partial rr}{\partial w}>0$, $\frac{\partial^{2}f}{\partial w^{2}}<0$). The student will then be advised to enter the place at another set time. The visualization of $f\left(w\right)$ and $g\left(p\right)$ is shown in Figure 1. Of course, these constants (10 min, 1 min, etc.) can be preliminary set by need. Thus, a possible mathematical model which corresponds to these requirements is defined as Equation (3). The process of handling the requests in Table 1 is shown in Table 2.

(3) $$rr\left(p,w\right)=\left\{\begin{array}{c} w+1,\;\;\;0<w\leq 10,\;\;\;0<p\leq 1\\ \left(w+p\right)/p,\;\;0<w\leq 10,\;\;\;p<1\leq 10\\ (w+p\log_{10}p)/p\log_{10}p,\;0<w\leq 10,\;p>10\\ 10\log_{10}w+1,\;\;\;w>10,\;\;\;0<p\leq 1\\ (10\log_{10}w+p)/p,\;w>10,\;\;p<1\leq 10\;\;\\ (10\log_{10}w+p\log_{10}p)/p\log_{10}p,\;w>10,\;\;p>10 \end{array}\right.$$

People are not lifeless computer programs. This queuing model has something in common with some process schedulers in operating systems. Notably, this model is designed for schools. It is mild and considers people’s willingness. When necessary, it can provide reasonable suggestions to users. For example, if a user’s request has been suspended for a long time, it means that perhaps there exist many people who are waiting. Therefore, the mechanism behind the mathematical model will make people give up.

### 3.2. Outdoor–Indoor Contact Tracing

In this section, we describe methods to obtain students’ locations and trajectories. Both outdoor and indoor environments are considered. An outdoor–indoor tracking model is established.

#### 3.2.1. Outdoor Tracing

**Figure 2:**
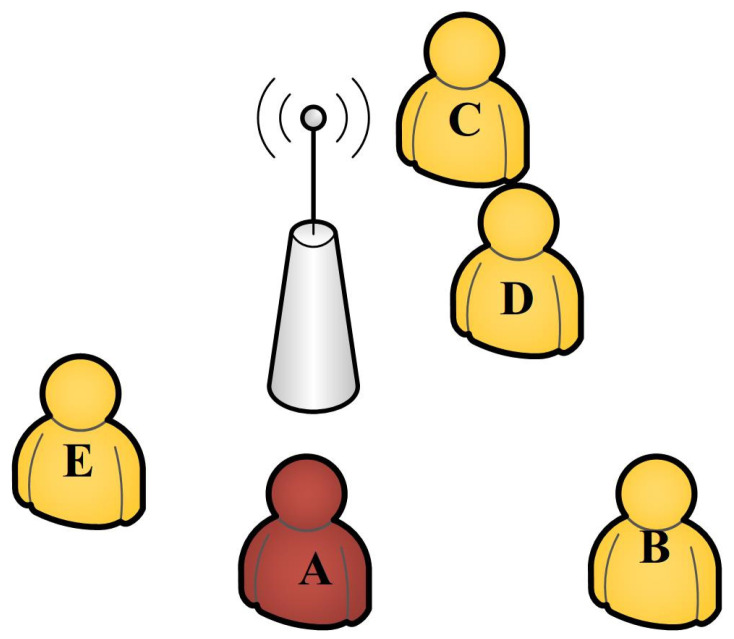
Using base stations to confirm potential infected people. The person in red is COVID-19-positive. Individuals in yellow are seen as risky.

There are many popular positioning methods in outdoor environments (satellite-based and base station-based), and these methods have already been put into use to track potential patients. A base station is a fixed transceiver which is the fundamental communication point for one or more wireless devices (e.g., smartphones). Base stations “know” which device is connected, and these data can be stored for further analysis. For example, person A’s (COVID-19-positive individual) mobile phone connected to base station A at 10:00, and during 10:00–10:30, B, C, D, and E also connected to the base station. This means that B, C, D, and E may have had contact with the patient (see Figure 2).

Nevertheless, station-based positioning is an approach with low positioning accuracy and large errors (sometimes even several kilometers) among several positioning methods with mobile phones. Schools are densely populated places. For example, there are about 40,000 people at Peking University, and the area is only about 2,500,000 m^2^. If, unfortunately, a student is infected, too many people will be involved. It is hard to take measures to test and quarantine these people very quickly. Actually, the risk of infection is not necessarily high for people connected to the same base station. A sparsely populated place may choose to use base stations to find potentially infected people. This simple method is hard to conduct in schools sometimes.

Therefore, satellite-based services can function effectively in solving this problem in outdoor environments. Normally, the mobile phone with a Global Navigation Satellite System (GNSS) could provide positioning services, and the errors are within several meters, which are already enough for tracking people. Obtaining the tracking and live positioning results of a person is important for the model to predict infection risk thereinafter.

#### 3.2.2. Indoor Tracing

However, these outdoor tracking methods are not appropriate in indoor environments. There are many buildings in schools. In one day, many people will go in and out of an enclosed space. If we find a COVID-19-positive person in them, we sometimes cannot immediately conduct nucleic acid tests for so many people and provide enough quarantine rooms. Therefore, we should know their tracking information in some buildings to obtain a deep analysis of individuals’ infection risk. A simple and cost-effective indoor positioning system (not indoor navigation) is required. We used a modified KNN algorithm and fingerprint-based database to tackle the problem.

Every location in an indoor environment is expected to have a unique fingerprint. For example, if temperatures in different positions are different, we can use a thermometer to predict the current location. However, this indicator is not commonly used. One possible indicator is the RSSI. The wireless technology (iBeacon node) is popular worldwide, which uses the 2.4 GHz band, and this band could be used freely. RSSI is an indicator to distinguish how well a mobile device could receive a signal from an AP. Normally, the greater the RSSI value, the better the signal. Devices supporting IEEE 802.11 can make the measurement available to users.

**Figure 3:**
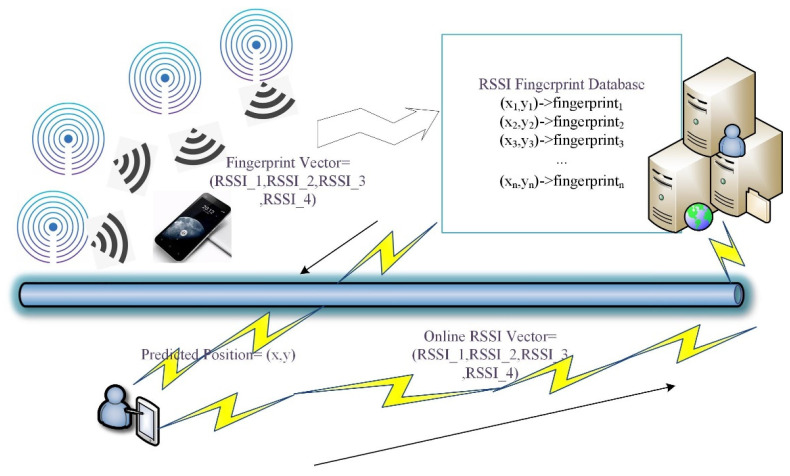
The general process of the establishment of an offline fingerprint database and online positioning.

A vector of RSSI values from a node can be represented as $r=\left(r_{1},r_{2},r_{3}\ldots r_{n}\right)$. These RSSI values can be measured by continuous measurements. In vector $R=\left(r_{1},r_{2},r_{3}\ldots r_{p}\right)$, $r_{t}$ is the “representative” RSSI value from node t. Then, we should select reference points and connect the vector R with the corresponding point to build a fingerprint database. For example, supposing there are 5 iBeacon nodes, an original record in the database is $\left(\left(1.3\;m,\;2.8\;m\right),\;\left(-43\;\mathrm{dBm},-44\;\mathrm{dBm},-50\;\mathrm{dBm},-70\;\mathrm{dBm},-80\;\mathrm{dBm}\right)\right)$. It means that the position $\left(1.3\;m,\;2.8\;m\right)$’s fingerprint is the vector. After the database is built, at every position, we can obtain a live RSSI vector from nearby iBeacon nodes. This live vector can be sent to the fingerprint database to obtain a predicted coordinate. The whole process is shown in Figure 3.

At every reference point, we can obtain many RSSI values from a node. These RSSI values are not the same. What we should do is find the most “representative” RSSI value. There are many methods to solve the problem. For example, the mean filter which is commonly used is defined as in Equation (4):

(4) $$r=\frac{1}{n}\sum_{i=1}^{n}r_{n}$$

This method is definitely easy to implement. In case the sample size is large, the RSSI fluctuation range is small, and the signal smoothness is high, it can yield a representative RSSI value; however, if RSSI values fluctuate greatly, the reliability of this method is very low. Using other simple methods such as the median filter, queueing filter, and Dixon filter cannot directly help to build a robust fingerprint database.

RSSI values from an iBeacon node can be seen as random variables, and $RSSI\sim N\left(\mu ,\sigma^{2}\right)$, where N means normal distribution. Therefore, the probability density function (PDF) of RSSI is denoted as:

(5) $$f\left(RSSI\right)=\frac{\exp\left(-\frac{{\left(RSSI-\mu \right)}^{2}}{2\sigma^{2}}\right)}{\sqrt{2\pi }\sigma }.\;$$

The average value, μ, is calculated by:

(6) $$\mu =\sum_{m=1}^{n}RSSI_{m}.$$

The variance, σ, is defined as:

(7) $$\sigma =\frac{1}{n-1}\sqrt{\sum_{k=1}^{n}{\left(RSSI_{k}-\mu \right)}^{2}}.$$

Let $0.6<F\left(RSSI\right)<1$, where $F\left(RSSI\right)$ is the cumulative distribution function (CDF). Thus, 0.15σ+u≤RSSI≤3.09σ+u, and RSSI values within the range $\left[0.15\sigma +u,3.09\sigma +u\right]$ will remain. Gaussian filter is capable of filtering some RSSI values that deviate from the ideal value. However, some interferences (e.g., shot noise) still influence the reliability of the representative RSSI value.

A simple unscented Kalman filter (UKF) can smooth a group of data with random errors to obtain more representative RSSI values and improve the robustness of the fingerprint database. It can be used in a non-linear system. We do not calculate the Jacobian and Hessian matrix, which is easy to implement [57]. The process is shown in Equations (8)–(17).

(1) The system is non-linear, and it is defined as:

(8) $$X_{k+1}=F_{k}\left(X_{k},\;U_{k},V_{k}\right),$$

and

(9) $$Y_{k}=H_{k}\left(X_{k},N_{k}\right).$$

(2) The system initial state is defined as:

(10) $${\hat{x}}_{0}^{a}=E\left(x_{0}^{a}\right)\left[{\hat{x}}_{0},0,0\right]$$

and

(11) $$P_{0}^{a}=diag\left(P_{0},Q,R\right).$$

The sampling point is defined as:

(12) $$\chi_{k-1}={\hat{x}}_{k-1}\;{\hat{x}}_{k-1}+\gamma \sqrt{P_{k-1}}\;{\hat{x}}_{k-1}-\gamma \sqrt{P_{k-1}},$$

where γ is the scale factor.

(3) Equations (13)–(15) are used for status prediction:

(13) $$\chi_{i}^{x}\left(k+1|k\right)=f[\left(\chi_{i}^{x}\left(k|k\right),u\left(k\right),\chi_{i}^{w}\left(k\right)\right]$$

(14) $$\hat{x}\left(k+1|k\right)=\overset{L-1}{\underset{i=0}{\sum }}\zeta_{i}^{m}\chi_{i}^{x}(k+1|k)$$

(15) $$\hat{z}\left(k+1|k\right)=\overset{L-1}{\underset{i=0}{\sum }}\zeta_{i}^{m}z_{i}(k+1|k)$$

(4) Observations and update, in Equations (16) and (17):

(16) $$W\left(K+1\right)=P_{xz}(k+1|k)P_{vv}^{-1}(k+1|k)$$

(17) $$\bar{x}\left(k+1|k+1\right)=\bar{x}\left(k+1|k\right)+W\left(k+1\right)\left(z\left(k+1\right)-\bar{z}\left(k+1|k\right)\right)$$

In the online positioning stage, let the predicted location be $l\left(x,y\right)$. $\left[F_{1}^{i},F_{2}^{i},F_{3}^{i}\ldots F_{n}^{i}\right]$ is an online RSSI vector, denoted as $f_{i}$. The distance between an online RSSI vector could be re-defined as Equations (18)–(20):

(18) $$W_{l}^{i}=\exp\left(\frac{{\left(x_{i}-x_{p}\right)}^{2}+{\left(y_{i}-y_{p}\right)}^{2}}{4\sigma^{2}}\right)$$

(19) $$D_{l}^{i}=\sqrt{\overset{n}{\underset{k=1}{\sum }}{\left(F_{k}^{i}-f_{k}\right)}^{2}}$$

(20) $$D_{l}^{i}{}^{\sim }=\frac{W_{l}^{i}*D_{l}^{i}}{\sum_{i=1}^{N}W_{l}^{i}}$$

The predicted coordinate can be calculated by Equation (21):

(21) $$(x_{given},y_{given})=\frac{\sum_{i=1}^{k}(\frac{1}{D_{l}^{i}{}^{\sim }}*l_{i})}{\sum_{i=1}^{k}\frac{1}{D_{l}^{i}{}^{\sim }}}$$

The symbol table of variables in these equations is presented in Table 3.

Generally, some parameters in the set of positioning algorithms cannot be confirmed using mathematical models in advance. Instead, they can be confirmed by real-world experiments. First, we can confirm the parameter k of the KNN algorithm. We can use different values of k to implement the algorithm, and by comparing these results, the value leading to optimal accuracy and stability will be chosen. After that, we should perform controlled experiments to distinguish the effect of data preprocessing and variants of the KNN algorithm. Finally, it is certain that students may move in indoor environments. Therefore, it is necessary to know if the positioning model is reliable when people are moving slightly.

However, we only know approximate locations and tracks through the fusion-positioning model. It is hard work to manually analyze these data. There are not enough computer engineers in a school who can directly observe these trajectory data. The problems of how to confirm every person’s risk level, and how to allocate resources in case of emergency, are preliminary solved hereinafter.

### 3.3. Risk Assesement and Resources’ Allocation

#### 3.3.1. An Approximate Algorithm to Define Contact Level

The algorithms and methods in this section are based on the following premises:

(1) Sometimes, COVID-19-positive individuals may not be isolated in time and they can freely hang out, especially asymptomatic patients [58].
(2) No matter whether COVID-19 patients have symptoms or not, they can infect other people.
(3) The farther away from a COVID-19 patient, the safer [59].
(4) The place where COVID-19 patients stay may be polluted. Even if the patient leaves, people who come to these places could still be infected [60].
(5) If a person has close contact with a COVID-19-positive patient, he will not be a new infection source very soon.

It is hard to propose an accurate model to precisely confirm everyone’s infection risk. People infected with different variants show different infectivity: some asymptomatic infected people do not infect others, people who are vaccinated may be less infectious, etc. Therefore, instead of an intricate probability model, we introduce a grid-based algorithm.

**Figure 4:**
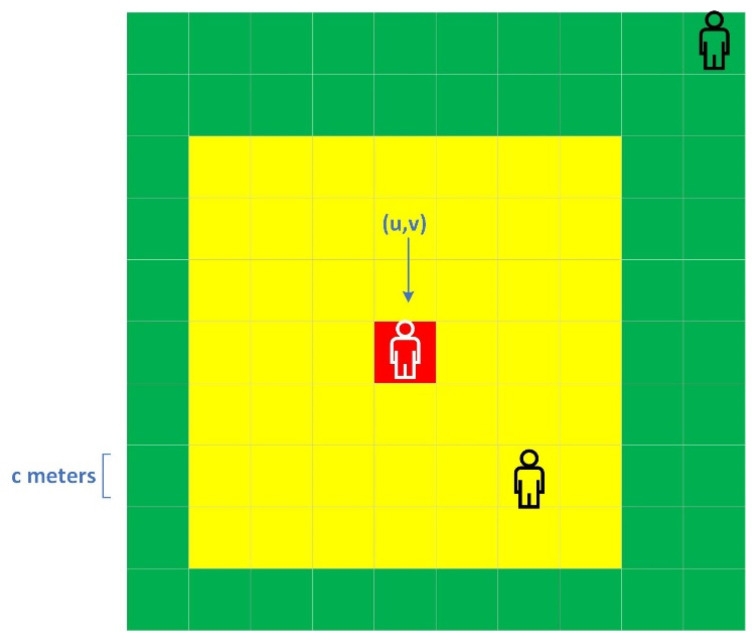
The position of the student in red is denoted as $\left(u,v\right)$.

A whole area can be divided into grids (c m* c m per one). In different environments, c can be different. A person’s real-time grid can be denoted as $\left(u,v\right)$, which is the index (coordinate) of the grid (see Figure 4). Another grid’s location is denoted as $\left(x,y\right)$. Other individuals’ contact levels relative to the person are confirmed by the grid where they stay (the coordinate $\left(x,y\right)$). For example, person P stays in $\left(0,0\right)$, and other people who stay in $\left(0,0\right)$ are seen as having close contact with P.

We then define COVID-19 contact in different levels (in Equation (22)). They are close contact, normal contact, and low contact. Of course, if a person never appears in the monitored area, he can be seen as no contact.

(22) $$\;Contact\;Level\;=\left\{\begin{array}{c} close\;contact,\;\;\;abs\left(x-u\right)=abs\left(y-v\right)=0\\ normal\;contact,\;\;\;0<abs\left(x-u\right)\leq 3,\;\;0<abs\left(y-v\right)\leq 3\\ low\;contact,\;\;\;other \end{array}\right.$$

However, Equation (22) only describes contact levels when all people stay still. People may move and contaminate objects in the environment. Let us suppose a person just directly vanishes from the whole area. If he is a patient, he has already contaminated the environment. The fact that others may still be infected even if a patient has left should also be considered in this model. Let t be the time from the disappearance of the person. The contact level can be confirmed by Table 4. The principle is that as t becomes larger, the contact level will be lower. When 2 h<t<6 h, another person in the grid can be seen as at the normal contact level. People will move in the area and we can obtain many results about contact levels. Additionally, a person’s contact level is influenced by results from Equation (22) and Table 4. The final risk level is the maximum value.

The parameters in the model can be adjusted according to need, as long as they are following the basic premises. For example, a larger c may make more people involved. Configuration should be adjusted to indoor and outdoor environments.

#### 3.3.2. The Use of Contact Levels

We have already described an approximate algorithm to define contact levels, and the parameters can be adjusted. After applying the algorithm, the contact level between every two people is confirmed. If a person is infected, we could then immediately know how many people are involved. If we find there exists a COVID-19-positive student, it is necessary to find out if others may be infected. Common ways are nucleic acid tests, antigen tests, computed tomography (CT), etc. However, medical resources are not enough in some regions. Thus, we should assign priority to testing those most likely to be infected, and then others, which is an avoidable compromise. It is much better than aimlessly making all people gather together to have nucleic acid tests. When we need to make potentially infected people isolate, tracking data can also be used.

**Figure 5:**
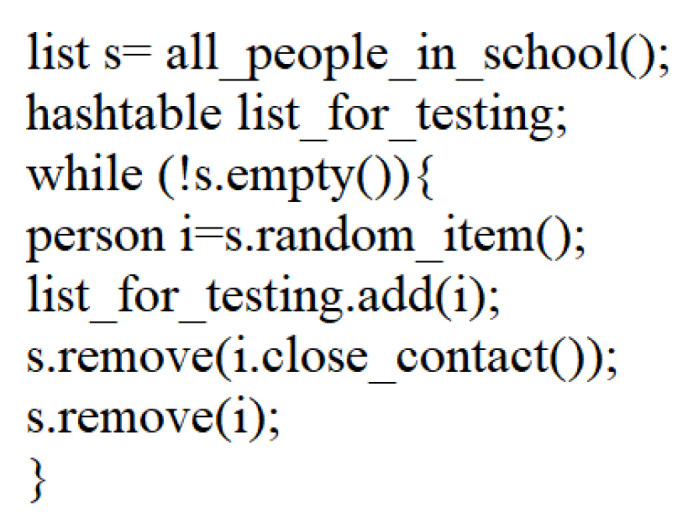
The pseudo code to identify infected people quickly.

**Figure 6:**
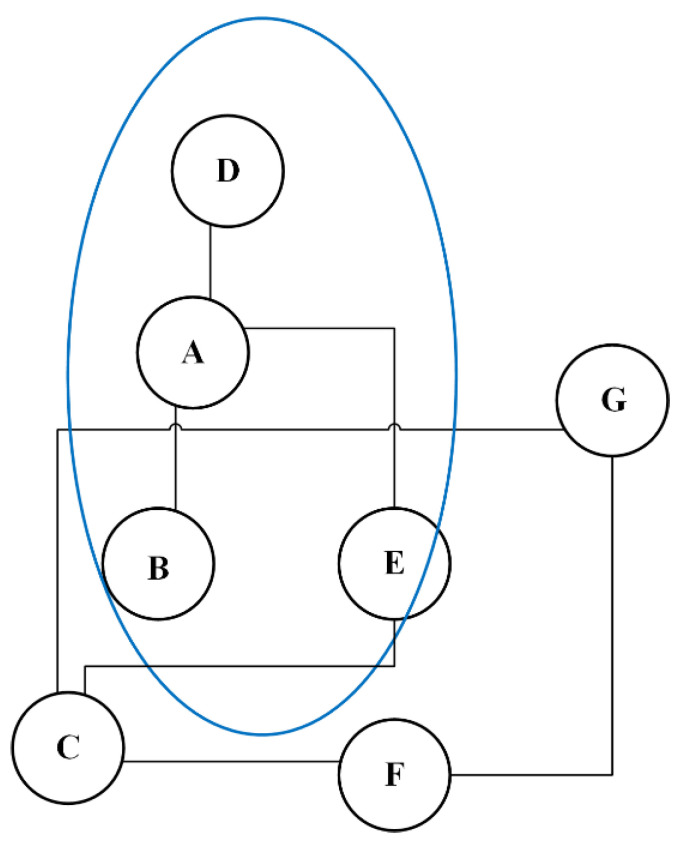
A’s close contact individuals are B, D, and E.

When there is a problem of finding the COVID-19 infection source, the tracking data can be used to quickly identify infected people. The pseudocode is shown in Figure 5. The main idea is that, if a person is healthy, potential contacted people are usually not infected. If a person is infected, people in potential contact are usually infected. In Figure 6, person A’s close contact people are B, D, and E. Thus, if A gets tests, D, B, and E can be removed from testing lists. Of course, if we can thoroughly test all people very soon, it is better.

In general, because of the limitation of resources, we should first allocate resources to people who have a higher probability of infection. This is much better than allocating resources aimlessly and randomly. The tracing data then play an important role.

## 4. Results and Discussion

The model is for COVID-19 control in school. Hence, effectiveness relies on the COVID-19 control effect and its cost. Schools using this model are expected to have lower infection risk, and lower costs, compared to harsh and one-size-fits-all solutions. The real-world infection risk test is not applicable at the present time. Since the principles to design the model are pandemic control rules which are demonstrated as valid by many scholars, we can test the effectiveness of components of the model instead.

### 4.1. Current Efforts for COVID-19 Control in School

Since the first COVID-19 case was confirmed (circa 2019), we have suffered great losses. Many people have known that SARS-CoV-2 is not a simple virus [61], although some infected people have mild symptoms, similar to influenza. The complexity and volatility of the COVID-19 pandemic challenge human beings: it is hard to predict the emergence of variants, some people who are vaccinated still become infected [62], asymptomatic infections are difficult to detect in time, etc. Due to the difficulties of tackling the pandemic, straightforward one-size-fits-all strategies are often conducted.

One-size-fits-all has two meanings. Both being blind to the pandemic and taking inflexible measures are seen as one-size-fits-all solutions. Although some people claim that COVID-19 is just a flu, which deserves no special attention, people are still making efforts to deal with the pandemic regardless of the effect [63]. A common flu can make many students unable to concentrate on learning in schools, let alone the more infectious and more serious COVID-19 pandemic. It is not a good idea to be blind to viruses spreading among students, especially vulnerable children. Some schools strongly recommend students and faculties to get vaccinated, some strictly check the itinerary of any person who attempts to enter the school, some regularly conduct nucleic acid tests on special faculties (e.g., canteen staff), etc. Since the outbreak of COVID-19, many technologies have been introduced to solve real-world problems, such as online learning [64], robot technology to combat COVID-19 [65], and smart wearable devices to monitor health conditions [66]. It means that we can make full use of various information technologies in the post-COVID-19 era to manage the schools better.

However, although we are making efforts to amend our policies and strategies, sometimes, we still conduct one-size-fits-all measures. Since social gatherings lead to a high infection risk, some affiliated canteens directly cancel eat-in food. It is hard to know the health conditions of people outside the school, so some schools ban students from going out de facto. Some school administrators forbid students from renting an apartment de facto, although living conditions in schools’ dormitories are sometimes bad, etc.

No matter how strict the one-size-fits-all strategy is, it can only be effective if the manager and the managed people cooperate well. The long-term implementation of these strict measures will lead to the slack of all individuals involved. It would be really awful if it caused the practice of formalities for formalities’ sake. Managers pretend that they are working hard for COVID-19 control, and people pretend to follow the rules. In this case, strict measures are nearly equivalent to no measures. Thus, we can see COVID-19 outbreaks in some schools, although they may have tried hard at prevention.

No model can guarantee to definitely stop viruses from spreading. However, we can follow the basic rules mentioned in epidemiology to control the pandemic. We do not intend to use state-of-the-art and intricate hardware, algorithms, and models. We instead focused on both social and technical feasibility.

### 4.2. The Feasibility of Positioning and Tracing

The reliability of contact level data is based on the indoor–outdoor positioning model. Therefore, in this section, we need to test static and dynamic stability.

As for the outdoor positioning system, there is no need to test it. GPS positioning systems and base stations are public infrastructure. They have already been demonstrated valid and feasible. Conversely, there are numerous devices and algorithms for indoor tracking. An optimized, cost-effective, and accurate indoor positioning system is required.

First, we need to select a reasonable k, which is an important parameter. If k is too large, the computation pressure is great, and sometimes it may cause too much noise. If k is too small, it is nearly a simple nearest neighbor (NN) algorithm, which may ignore useful information. We deployed the indoor positioning system in an indoor environment (20 iBeacon nodes evenly distributed, valid size 18 × 15 m, 120 reference points).

**Figure 7:**
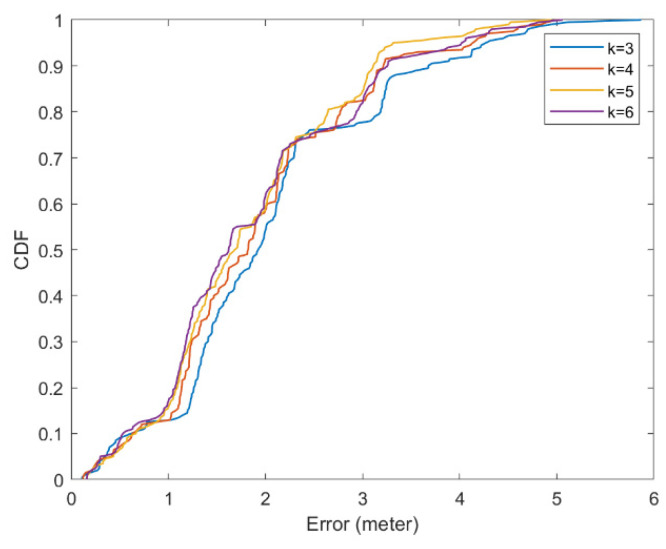
Positioning errors of different values of k (CDF).

With different values of k, we conducted static positioning tests. We selected 20 reference points, and at each point, we obtained 10 positioning results. The results are shown in Figure 7. The average positioning errors were 2.07, 1.93, 1.84, and 1.87 m, respectively. Thus, we used k=5 in the following tests.

**Figure 8:**
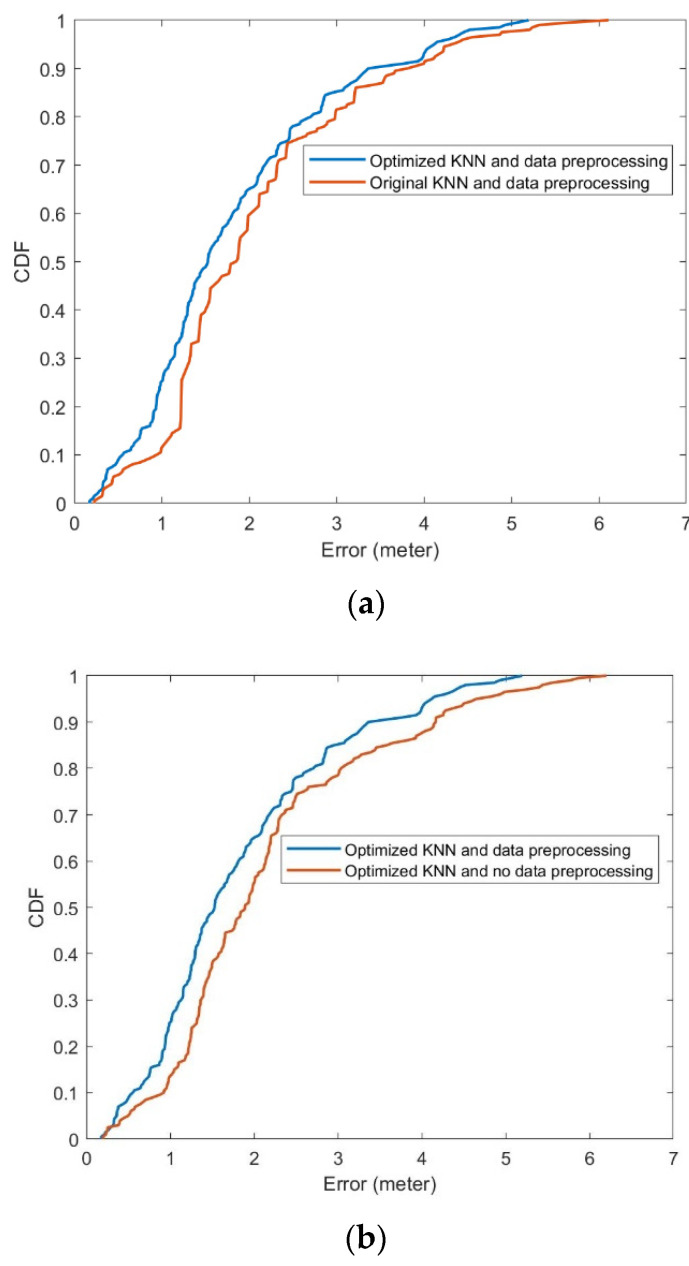
(**a**) Optimized KNN and data preprocessing, original KNN and data preprocessing. (**b**) Optimized KNN and data preprocessing, optimized KNN and no data preprocessing.

Then, to test the static stability of positioning, we carried out 200 positionings at a fixed point $\left(9,7\right)$. In addition, to demonstrate the effect of data preprocessing, controlled experiments were also performed. In Figure 8, the overall effect of data preprocessing and the optimized KNN, effect of data preprocessing, and effect of the optimized KNN are presented. Original KNN means we just used the original Euclidean distance between two vectors. No preprocessing means we just used a preliminary mean filter to obtain a representative RSSI value. The detailed data are shown in Table 5.

**Figure 9:**
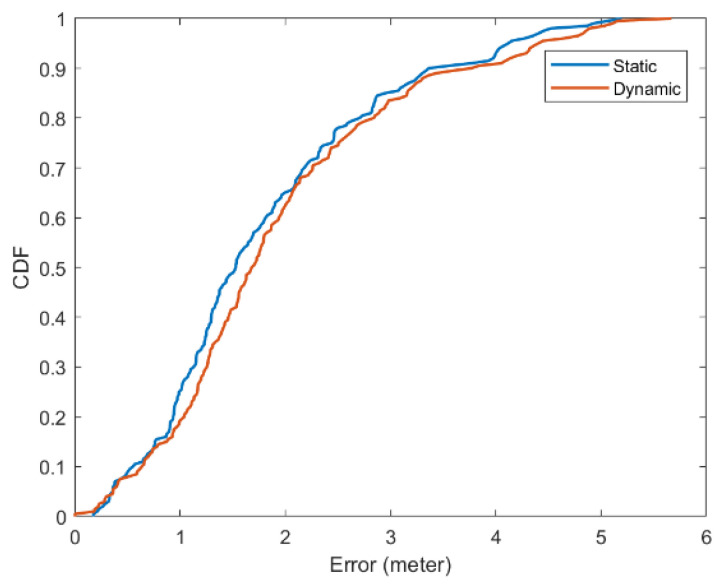
Errors when people stay still (static), and when people move (dynamic).

People’s movement may also influence the stability of positioning. Therefore, we recorded errors while moving. One of us simulated as a user and walked from point $\left(1,1\right)$ to $\left(10,10\right)$ and then came back over and over again, until we obtained 200 positioning errors. We obtained a predicted coordinate every 2 s. Although movement of individuals may affect accuracy, the positioning results are generally reliable. The details are shown in Figure 9 and Table 6. The CDF figures are plotted with the support of Matlab R2021a. Built-in plot-related and CDF-related functions can produce figures.

Compared to current state-of-the-art indoor positioning models [67,68,69], the performance and accuracy may not be better. However, we should consider the actual situation of schools. The more complex the model, the higher the maintenance cost. For example, if we plan to deploy guiding robots in a museum, the accuracy and requirements are high, and to improve users’ experience, a higher budget is acceptable. Instead, for schools, we should use cheap and effective measures, as long as it meets the need. We do not need to spend a lot increasing the accuracy by a few centimeters. iBeacon nodes are cheap and easy to deploy. They do not need an extra power supply. After an iBeacon node is placed well, we do not need to always pay special attention. The optimized KNN algorithm is easy to implement, and it requires no explicit training stage, which (training stage) is mandatory when using SVM, MLP, LSTM, etc. The results presented have already demonstrated the performance of the positioning system. In general, it meets our needs and is easier to popularize compared to other models.

### 4.3. The Possibility of Promotion

Nowadays, different countries have adopted different COVID-19 control policies. Many countries (e.g., Australia) have abandoned zero-COVID policies. It does not mean they have given up on COVID-19 control. Some other measures, such as large-scale vaccination programs, are seriously considered [70]. Indeed, it is very hard to conduct zero-COVID policies. Many factors, such as people’s willingness, the economy, and laws, will significantly influence the authorities’ decisions. For example, if a country’s economy depends heavily on foreign trade, it is unacceptable to block goods from foreign countries all the time. Otherwise, serious social problems (e.g., financial ruin) will also lead to casualties. Certainly, the virus can kill or harm people, but inappropriate policies and strategies can also cause severe problems, including casualties not directly related to virus infection.

There also exist countries (e.g., China) persisting with zero-COVID. In this section, we do not intend to discuss the pros and cons of zero-COVID or non-zero-COVID policies. Every country has the right to make decisions according to its own circumstances. We deem that no matter whether governments adopt the zero-COVID or non-zero-COVID policy, their attitude towards pandemic prevention and control is positive. Therefore, any solution which intends to tackle COVID-19 has the potential value to be promoted.

Any such implementation of the technology-involved program as this paper describes would lead to many costs. It is not only limited to financial costs, but also human resources, time spent, and a number of management efforts to persuade people to become used to the solution. In fact, many schools have already spent a lot on COVID-19 and we have already suffered many losses [71]. It is a big challenge for the authorities to manage a school well in the post-COVID-19 era.

People are making various efforts at COVID-19 control, but usually, many measures are manually conducted. Many individuals are involved, and actions are not precise. Therefore, digital transformation in public health, which is also mainstream nowadays, can play a significant role in reducing costs [72].

There exist various strict measures in schools to control social gatherings, but they lack scientific management, which sometimes leads to a mess. We can still see a throng of people rushing into a place (e.g., the canteen). Even if they may be aware that it is not good to gather together in the pandemic era, a single individual cannot urge others to follow the rules. There are some methods to implement the social gathering control model. For example, in a canteen, before entering, people should scan the QR code to be in the queue. When their requests are satisfied, they are permitted to enter the area. In the whole process, an indoor positioning system can function effectively in finding out whether a person follows the queuing rule.

In indoor environments, the risk of infection is relatively higher. Therefore, indoor tracking is also necessary, especially for some large indoor spaces in which many students go in and out every day. Hardware and algorithms for indoor tracking are not complex or expensive. The parameters in risk assessment and resources’ allocation are different for indoor and outdoor spaces. Using different parameters will involve a different number of people. Managers should consider their medical resources and abilities. They can be setup by managers according to current situations.

To sum up, this paper was not intended to propose brand new theories in computer science. We focused on the design of the model to solve real-world problems: management and decision-making during the long-lasting pandemic. We have introduced various technologies in order to implement COVID-19 control measures in schools in a scientific and user-friendly way. It is a human-centered and social-technology model, which does not only consider technologies, but also society and humans.

## 5. Conclusions

This paper presented a sustainable management model for COVID-19 control in schools, which can: (i) control social gatherings, (ii) track individuals in indoor and outdoor environments, and (iii) assess people’s risk level and allocate medical resources in case of emergency. It makes improvements to one-size-fits-all strategies, improving management efficiency and lowering people’s pressure. We did not focus on developing brand new theories in information technology.

The scientific evidence to design the model is very clear. General methods in medicine to prevent infectious diseases are: cutting the transmission route, protecting vulnerable individuals, and controlling the infection source. They are highly interrelated, and we should consider every aspect of them in the model. The positioning system plays a vital role in the model. The iBeacon technology with fingerprint-based algorithms was introduced. The stability and accuracy (1.82 m when people stay still, and 1.95 m when people move) met the requirements. Data from the positioning system can be exploited for risk assessment and resources’ allocation. The simplified risk assessment method considers two dimensions: time and space. Medical resources can be preferentially allocated to people who are at higher risk in case of emergency. The implementation of social gathering control could also make full use of the indoor positioning system. It can guarantee and monitor people’s status. Only when the proposed methods cooperate well can the goal be achieved.

Appendix A provides a brief overview of this paper. The novelties of this paper are:

(1) We used a customized queue model to manage the throng of people to avoid social gatherings, which is hard to see in real-world school management.
(2) We considered the high infection risk in indoor environments, and indoor tracking technology was introduced.
(3) We used a simplified model to assess people’s contact levels. Based on these indicators, we can allocate resources more effectively compared to using random and aimless strategies.
(4) The proposed model is feasible both in technology and society.

## 6. Limitations and Future Work

The model follows principles in epidemiology in general. Components of this model have been demonstrated as valid using theoretical analysis and in-field experiments. However, the overall evaluation is missing. This can only become clear when conducting controlled experiments. If some schools want to consider the model, and others do not, we can find out the overall effect. Then, based on these data, we can perform further optimization. The infection rate is expected to be lower and normal life should not be excessively hindered.

In addition, data privacy protection measures of the model are necessary. There are many data exchanges when people are engaged in the model. Thus, there is a risk of privacy leaks. This topic can be further elaborated on in the future.

## Figures and Tables

**Table 1:** The situation where 4 people intend to enter a canteen which can only contain 1 person.

Student Name	*Request Time*	*Predicted Staying Time*
A	10:20	10 min
B	10:25	10 min
C	10:30	15 min
D	10:31	5 min

Request Time is the moment when a person applies to enter the canteen. Predicted Staying Time is an estimated value, indicating how long a student will stay in that area. The values can be predicted using statistical data.

**Table 2:** The statues of requests. “Not applicable” means that someone has not arrived or left. “Yes” means a person has entered the canteen with permission already. “Waiting” indicates that a student’s request cannot be immediately satisfied. Note that not every moment is listed.

Time	Student A	Student B	Student C	Student D
10:20	Yes	Not applicable	Not applicable	Not applicable
10:25	Yes	Waiting	Not applicable	Not applicable
10:30	Not applicable	Yes $(rr_{B}>rr_{C}$)	Waiting	Not applicable
10:40	Not applicable	Not applicable	Waiting	Yes $(rr_{D}>rr_{C}$)

**Table 3:** The symbol table of variables which are used to predict a location.

Name	Annotation
$D_{l}^{i}$	Euclidean distance
N	Number of reference points
σ	Maximum possible distance of going from previous position to l within the sampling interval
l	Predicted location
$\left(x_{p},y_{p}\right)$	p means previous

**Table 4:** The strategies for changing contact levels when a person disappears.

Time	Close Contact	Normal Contact	Low Contact
0<t≤1 h	close contact	normal contact	low contact
1 h<t≤2 h	close contact	low contact	low contact
2 h<t≤6 h	normal contact	low contact	low contact
t>6 h	low contact	low contact	low contact

**Table 5:** Detailed information of controlled experiments.

	Data Preprocessing and Optimized KNN	Data Preprocessing and Original KNN	No Data Preprocessing and Optimized KNN
Less than 0.5 m	8.5%	5.5%	5%
Less than 1.5 m	48.5%	39.5%	37.5%
Less than 2.5 m	78%	74.5%	73.5%
Less than 5.0 m	99%	97.5%	96.5%
Average Error	1.82 m	2.07 m	2.15 m

**Table 6:** Detailed information of errors when people move and stay still.

	People Stay Still	People Move
Less than 0.5 m	8.5%	7.5%
Less than 1.5 m	48.5%	41.5%
Less than 2.5 m	78%	74%
Less than 5.0 m	99%	98%
Average Error	1.82 m	1.95 m

## Data Availability

Not applicable, as the study did not report any data.

## Appendix A

Here is a quick Q&A index which can help to understand the main idea of this paper. All these problems were already answered in the main text.

Q1: Is this model necessary for schools?

A1: Yes.

We should pay special attention to COVID-19 control. Although zero-COVID policies are very hard to conduct, we should not be blind to viruses spreading among students.

The persistent inflexible and strict policies often make people exhausted. Thus, sometimes managers pretend that they are working hard, and people pretend that they do follow the rules. This model can play a role in improving management efficiency and lowering people’s pressure.

Q2: Why not use the xxx algorithm/model/device?

A2: As mentioned in the main text, we do not intend to create brand new theories in computer science. To address our purpose, we introduce and optimize these necessary technologies. Both social and technical feasibility are considered. A complex and costly model may make school managers less interested, and in that case, the design can only exist on paper.

Q3: Why does the contact level algorithm ignore some influence factors, for example, types of viruses?

A3: There are many influence factors, such as types of viruses, temperature, people’s health condition, etc. Considering too many factors will lead to extreme complexity and put forward higher requirements for the accuracy of parameters, which often means less robustness. The algorithm is based on five basic premises.

Q4: Compared to the current status, what improvements are made?

A4: First, a customized queue model has been introduced. The school managers ask people to maintain social distance and intend to control social gatherings, but we cannot usually see these scientific measures. Then, contact tracing is more precise, and based on tracing data, we can make decisions more accurately, which will not involve too many people, so that the costs (human resources, time spent, investments, etc.) will be lower.

Q5: What is the scientific evidence to create the model?

A5: Three principles in epidemiology: cutting the transmission route, protecting uninfected people, and controlling infection sources.

Q6: What are the authors’ general attitudes towards current strategies and policies?

A6: Schools’ COVID-19 prevention and control measures should follow local laws first. We appreciate everyone working hard for COVID-19 control.

We stay neutral with regard to any policy and strategy. Our purpose is to contribute to COVID-19 prevention and control, not to criticize or praise any strategy and policy.

We seriously emphasize that all discussions are for academic purposes only.
